# Supplementary figures and images for: Generation of targeted homozygosity in the genome of human induced pluripotent stem cells
Source: PLoS One. 2019 Dec 5;14(12):e0225740. doi: 10.1371/journal.pone.0225740 (PMC6894808; doi:10.1371/journal.pone.0225740)

**A**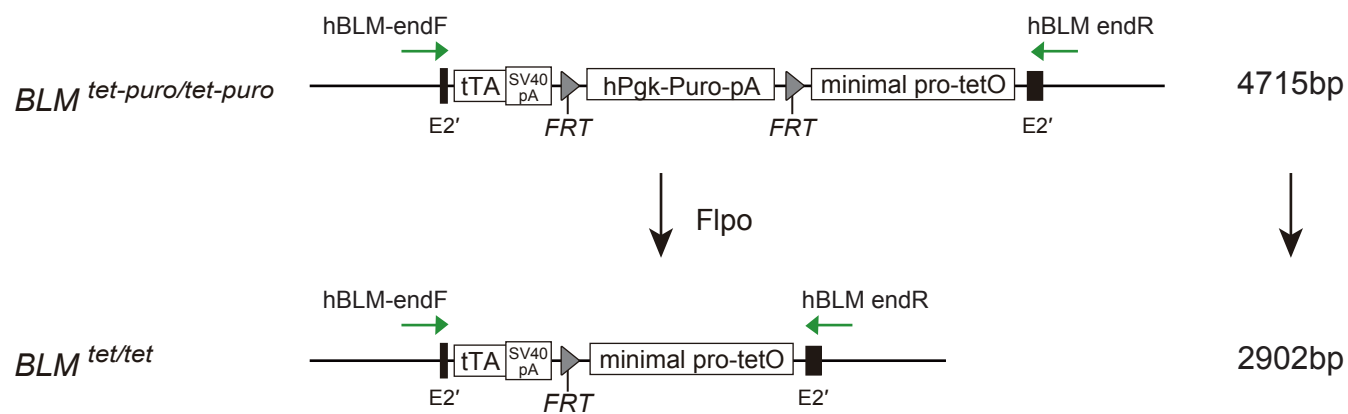**B**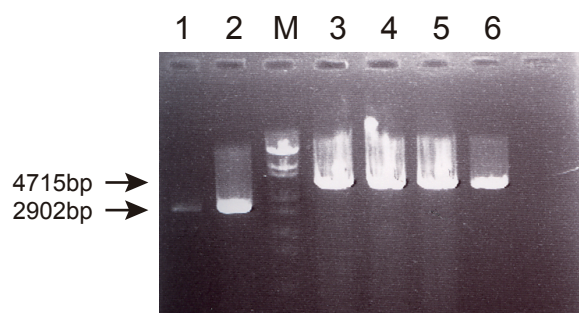

Supplement: S1 Fig — (A) Schematic representation of the BLM locus before and after removal of the selection cassette. Primers hBLM-endF and hBLM-endR were used to detect the removal. (B) Analyses of colonies after Flpo transfection by PCR with primers hBLM-endF and hBLM-endR. The selection cassette was deleted in clones #1 and #2, but not in clones #3–#6. M indicates the marker. (PDF) [file pone.0225740.s001.pdf]

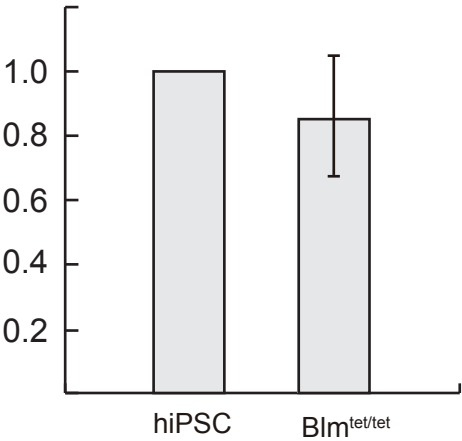

Supplement: S2 Fig — FPKM derived from hiPSC-BLMtet/tet was divided by that from hiPSCs. (PDF) [file pone.0225740.s002.pdf]

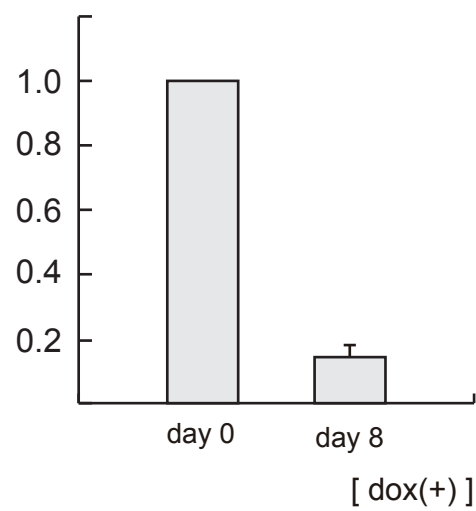

Supplement: S3 Fig — The method to detect BLM transcripts was the same as that in Fig 1D. (PDF) [file pone.0225740.s003.pdf]

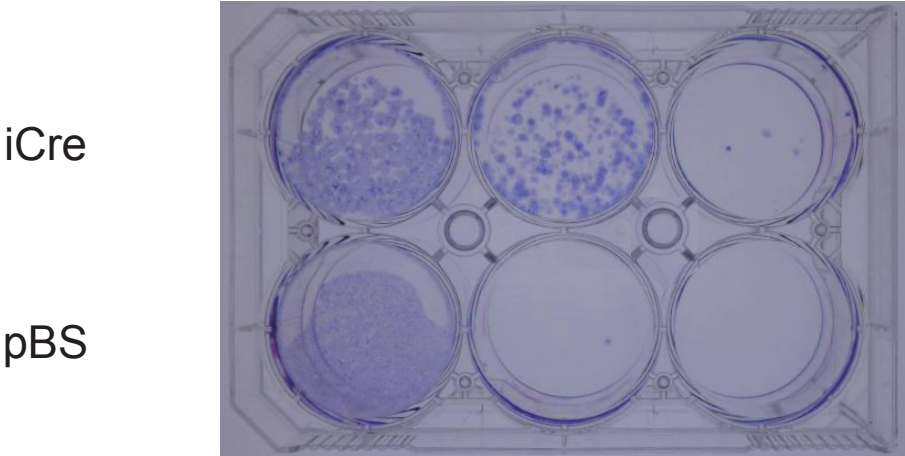

|                  |   |   |   |
|------------------|---|---|---|
| G418 (100µg/mL)  | + | - | + |
| Puro (0.75µg/mL) | - | + | + |

Supplement: S4 Fig — hiPSC-BLMtet/tetAAVScNP/+cells (5×10^5^) were transfected with EF1α-iCre or pBluescript (pBS), followed by selection with either 100 μg/mL G418, 0.75 μg/mL puromycin, or both. The efficiency of Cre-mediated cassette inversion was estimated at approximately 10 days after selection. Surviving colonies after the selections were fixed and stained with Giemsa stain. They are shown with blue dots. (PDF) [file pone.0225740.s004.pdf]

**A**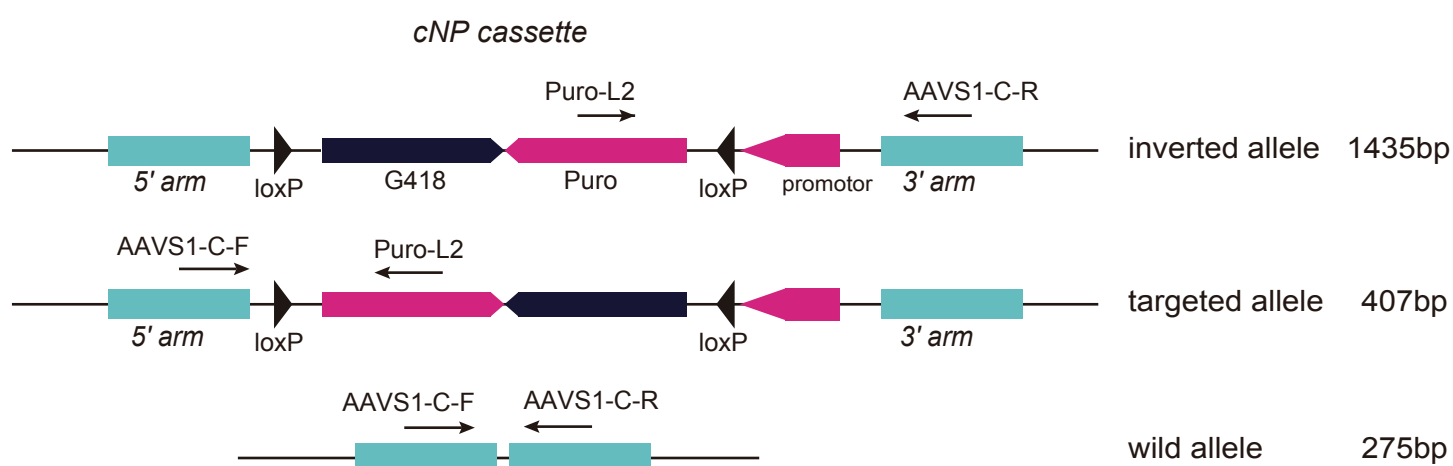**B**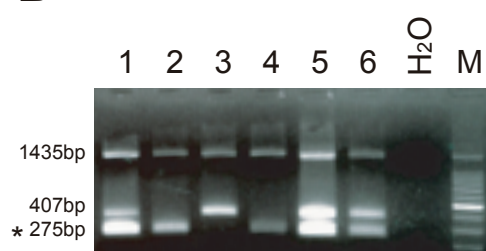

Supplement: S5 Fig — (A) Candidate clones were analyzed by competitive PCR using primers AAVS1-C-F, AAVS1-C-R, and Puro-L2 (S1 Table). hiPSC colonies after selection were picked up and applied to PCR analysis or culture in 96-well plates. (B) Wildtype, neomycin resistance, and puromycin resistance alleles are represented by bands of 275, 407, and 1435 bp, respectively. The band derived from the wildtype allele is indicated by an asterisk. The positive pattern losing the band derived from the wildtype allele is shown in lane 3. M indicates 100 bp markers. (PDF) [file pone.0225740.s005.pdf]

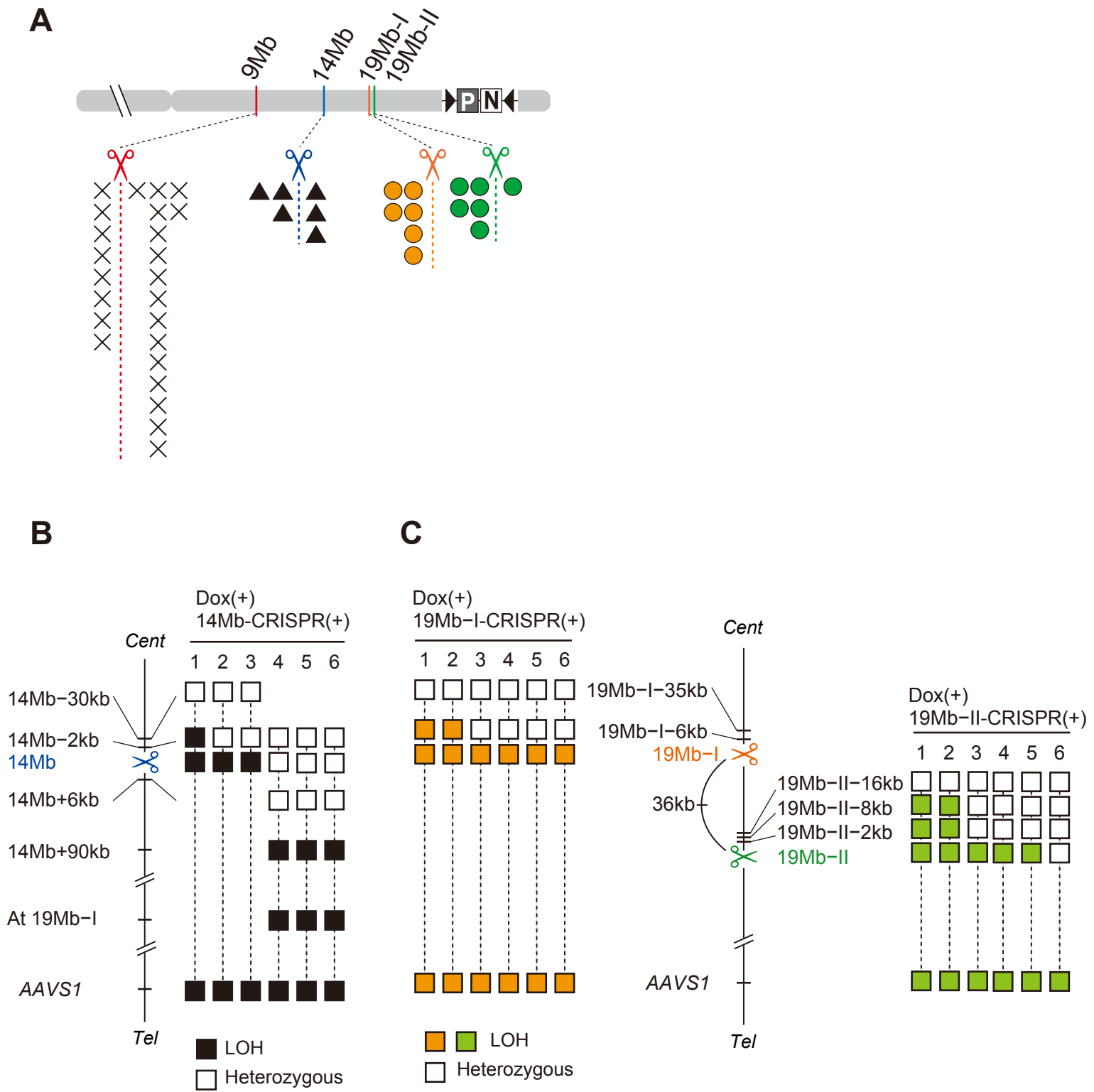

Supplement: S6 Fig — (A) Analyses of crossover points in chromosome 19 (9Mb, 14Mb, and 19Mb). (B) LOH panel of crossover points at 14 Mb. (C) LOH panel of crossover points at 19 Mb-I and 19 Mb-II. (PDF) [file pone.0225740.s006.pdf]

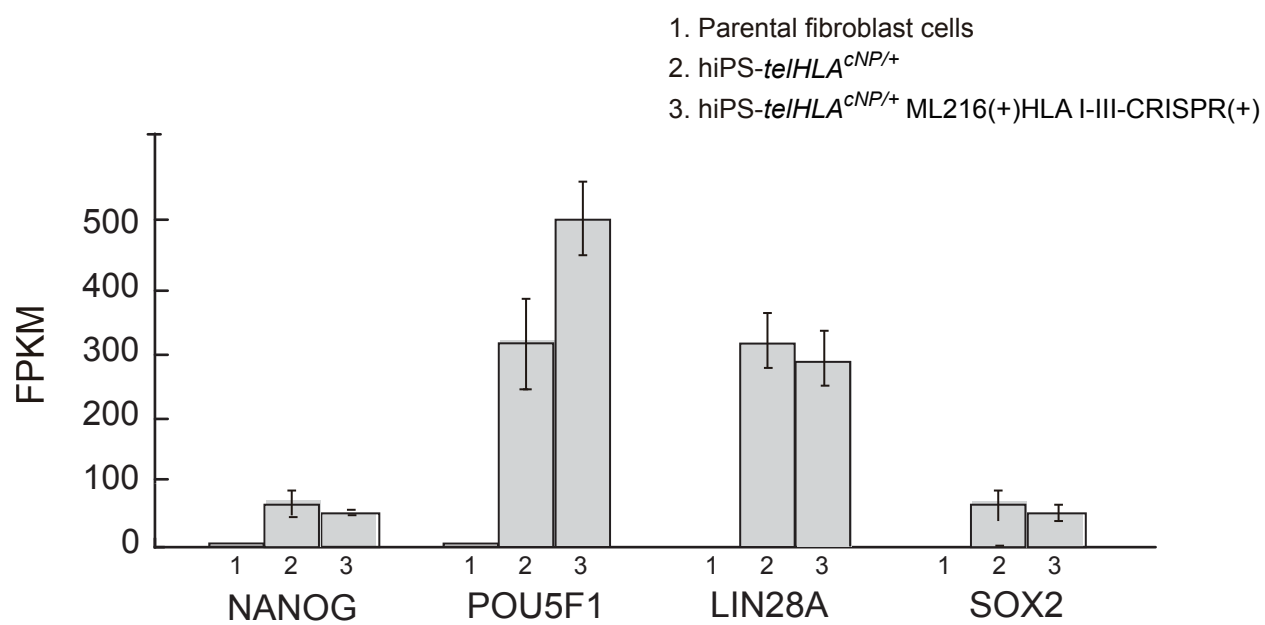

Supplement: S8 Fig — FPKM (fragments per kilobase of exon per million mapped reads) was calculated from total RNA of parental fibroblasts (1), hiPSC-telHLAcNP/+ (2), and hiPSC-telHLAcNP/+ML216(+)HLA I-III-CRISPR(+) (3). (n = 3, error bars show SEM). (PDF) [file pone.0225740.s008.pdf]

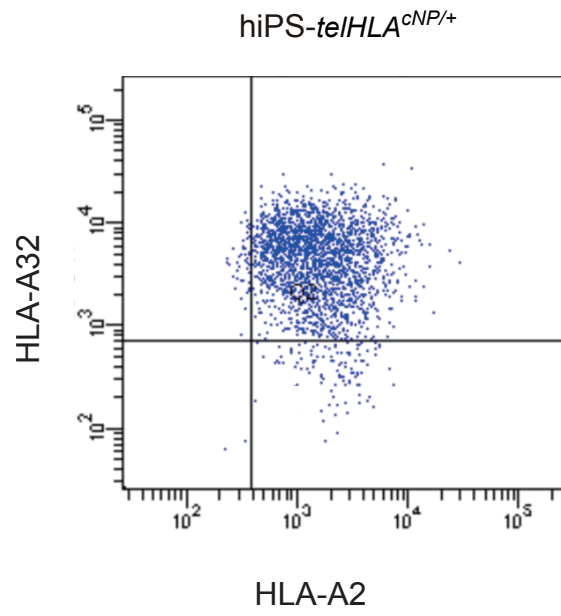

Supplement: S9 Fig — (PDF) [file pone.0225740.s009.pdf]
