# Supplementary material for: Generation of targeted homozygosity in the genome of human induced pluripotent stem cells
Source: PLoS One. 2019 Dec 5;14(12):e0225740. doi: 10.1371/journal.pone.0225740 (PMC6894808; doi:10.1371/journal.pone.0225740)

S7 Fig

Parental fibroblast cells

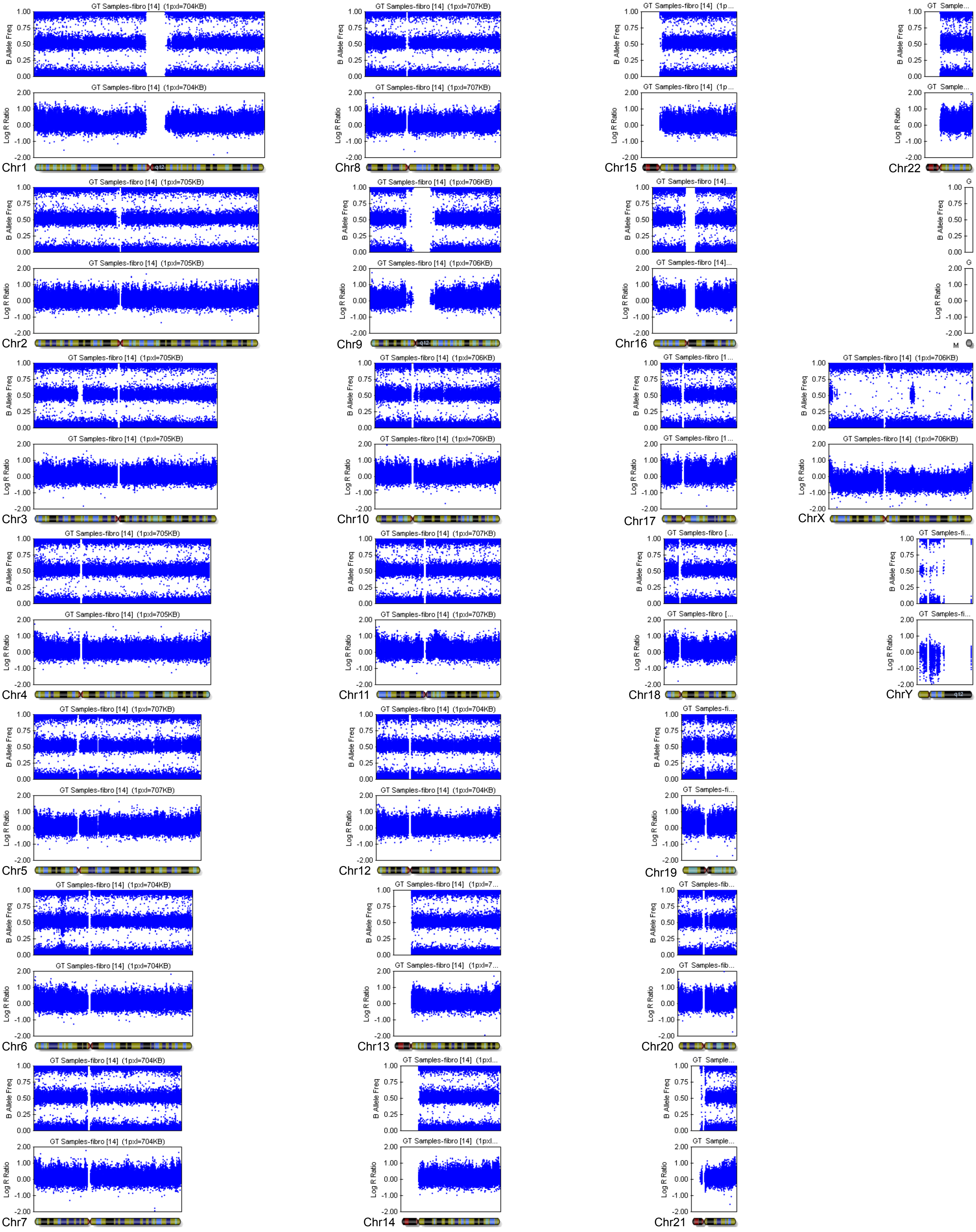

hiPSCs

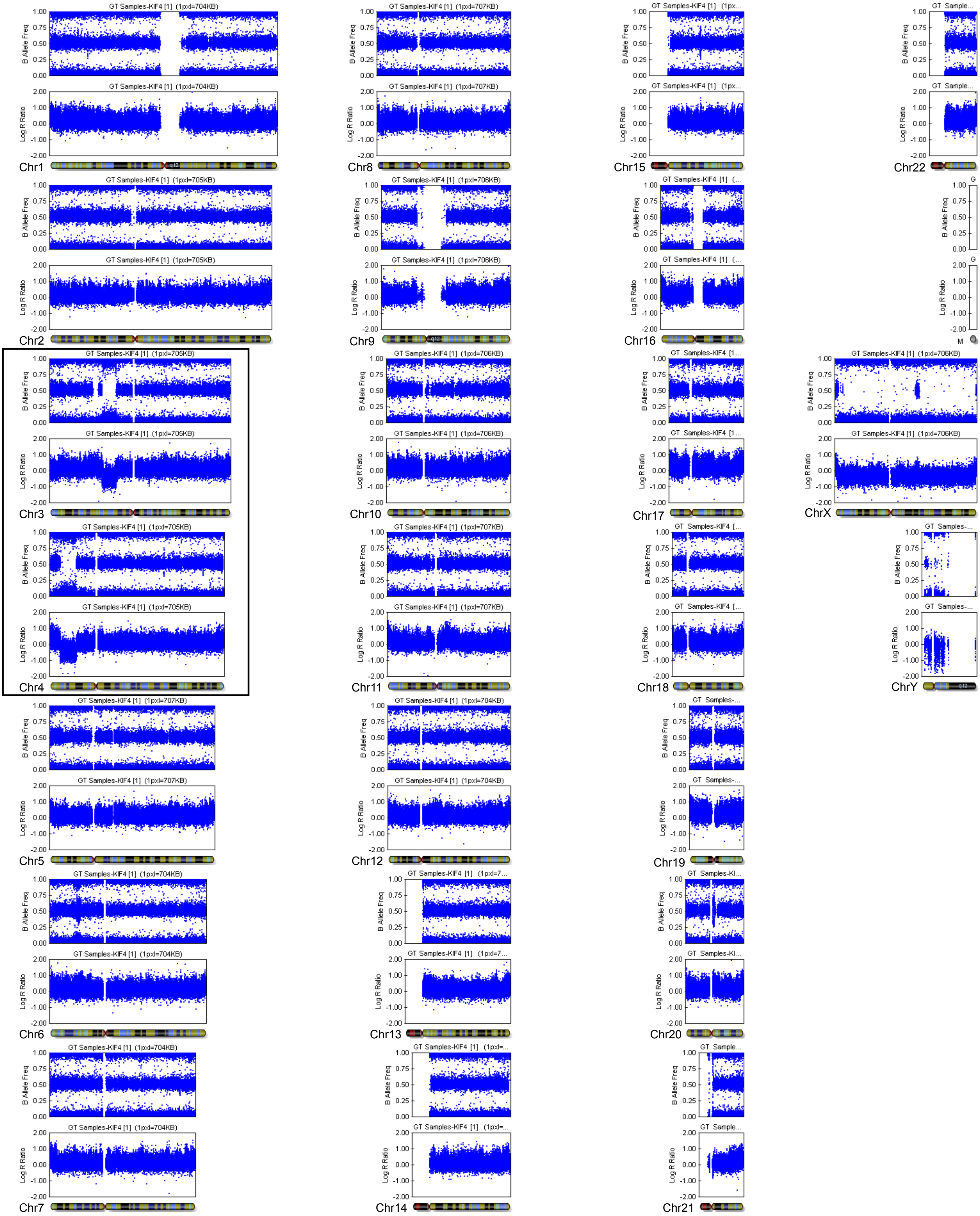
GT Samples-KIF4 [1] (1pxl=704KB)
B Allele Freq
Log R Ratio
Chr1
Chr2
Chr3
Chr4
Chr5
Chr6
Chr7
Chr8
Chr9
Chr10
Chr11
Chr12
Chr13
Chr14
Chr15
Chr16
Chr17
Chr18
Chr19
Chr20
Chr21
Chr22
ChrX
ChrY

hiPSC-*BLM*$^{tet/tet}$*AAVS*$^{cNP/+}$ Dox(+) 9M-CRISPR(+)

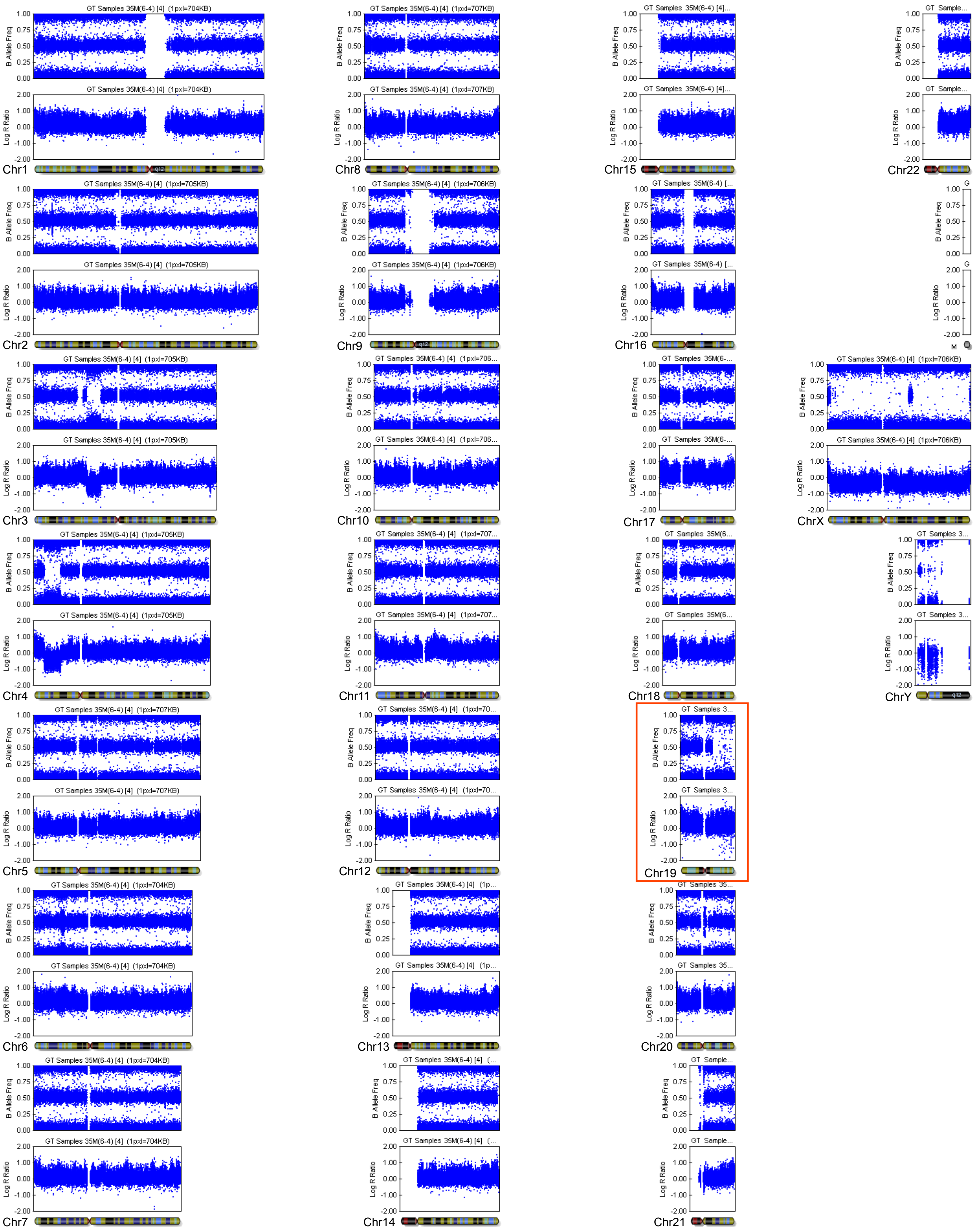

S7 Fig : Continued

hiPSC-*BLM*$^{tet/tet}$*AAVS*$^{cNP/+}$ ML216(+) 9M-CRISPR(+)

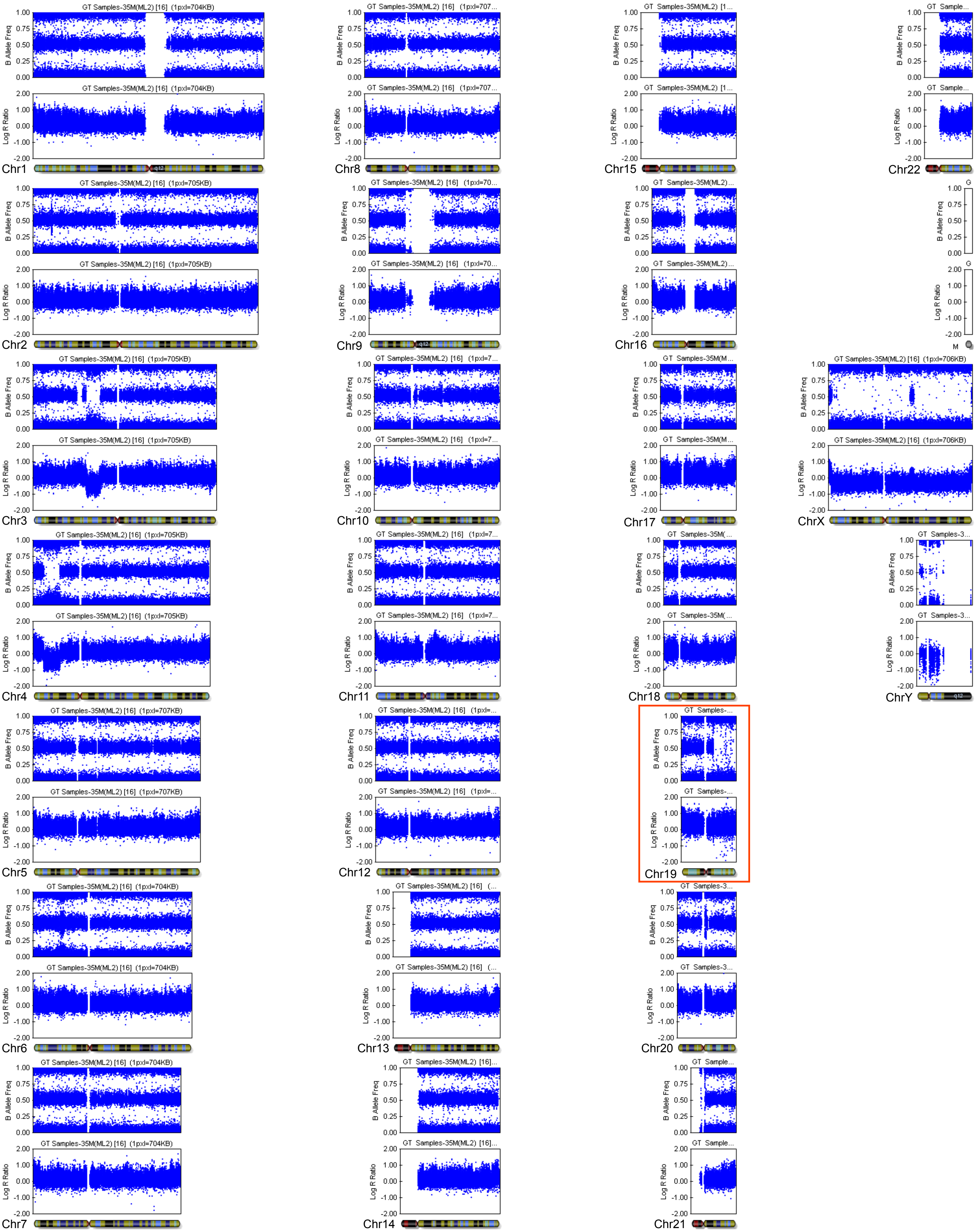

Supplement: S7 Fig — SNP array patterns were the same in hiPSCs and two clones [hiPSC-BLMtet/tetAAVScNP/+DOX(+)9M-CRISPR(+) and hiPSC-BLMtet/tetAAVScNP/+ML216(+)9M-CRISPR(+)] after crossovers except in predicted sites (red box). hiPSCs had abnormal chromosomes 3 and 4 as indicated by a black box. These abnormalities might have occurred during hiPSC generation, because parental fibroblasts did not contain these abnormalities. (PDF) [file pone.0225740.s007.pdf]
