# Supplementary material for: Generation of targeted homozygosity in the genome of human induced pluripotent stem cells
Source: PLoS One. 2019 Dec 5;14(12):e0225740. doi: 10.1371/journal.pone.0225740 (PMC6894808; doi:10.1371/journal.pone.0225740)
Supplement: S1 Table — (PDF) [file pone.0225740.s010.pdf]

S1 Table. Primers used for vector construction and PCR genotyping

Vector construction

Usage	Primer name	Primer sequence
BLM targeting arms	hBLMLarmF1	TCTGTTTAAACAAGATCAATACTTTTAAACTGGATTCCAAA
	hBLMLarmR1	GGCTTAATTAAATAATCCTAAAAAGTGAGGGAAAAAGAAAT
	hBLMRarmF1	ATCTGCGGCCGCGATTATGGCTGCTGTTCCTCAAAATAATCT
	hBLMRarmR3	AGAAGGTGGAACAAAATCCGTATCATAATC
	hBLMR endF	TCCTATTACTCTGGGCACAGTTGGAACAAT
	hBLMR endR	TGGTAAGTTAGTGCCTATGTGACAGCAAAC
AAVS1 targeting arms	AAVS1-LF1	ATATGTTTAAACGAGCCAGGGGCATGAGATGGTGGACGAGGA
	AAVS1-LR1	ATATGGCGCGCCCACTAGGGACAGGATTGGTGACAGAAAAGC
	AAVS1-RF1	ATATGGCCGGCCGCCCCACTGTGGGGTGGAGGGGACAGATAA
	AAVS1-RR1	ATATATTTAAATTTCTCTGACCTGCATTCTCTCCCCTGGGCC
Telomeric region of Chr6 targeting arms	chr6-Asc1-up	GGGCGAATTGGGGCGCGCCGGACAAAGCCACTGAAGGAGAGTAGG
	chr6-Asc1-low	TAATCTAGAATGGCGCGCCGTGGCGGCTATTTTCTCTCCTACAGC
	chr6-Pac1-up	GTATGCGGCCGCTTAATTAATTAAAGGACTGCAGGAAAAACAGGGTC
	chr6-Pac1-low	ACAAAAGCTGGATTAATTAATTGGACTGCCACCTTGCAGAGGGAGC

Screening for targeted clone

Usage	Primer name	Primer sequence
BLM locus	hBLM-1	TCCTATTACTCTGGGCACAGTTGGAACAAT
	hBLM-2	TGGTAAGTTAGTGCCTATGTGACAGCAAAC
	tTA-1	GTATGCCGCCATTATTACGACAAGCTATCG
	hBLM-3	AAGTGGGACAAAAGCTGTATTATGATCATG
	tTA-2	GTGAGTATGGTGCCTATCTAACATCTCAAT
AAVS1 locus	AAVS1-LF2	GATGCAGGGGAACGGGGCTCAGTCTGAAGA
	Puro-L2	GCGAGGCGCACCGTGGGCTTGTACTCGGTC
Telomeric region of Chr6	chr6-TG-low	CATGCAGCAAAATCCTTTATATTTTACATC
	chr6-TG-up	GAGCTGGAGCTACTGGTAATGACAAGGTC
	hEF1a-TG	GGATCAAGAATCACGTACTGCAGCCAGGTG

Screening for double positive clone

Usage	Primer name	Primer sequence
Chromosome 19	AAVS1-C-F	ACTAGGAAGGAGGAGGCCTAAGGATGGGGC
	AAVS1-C-R	GCTCTTCCAGCCCCCTGTCATGGCATCTTC
	Puro-L2	GCGAGGCGCACCGTGGGCTTGTACTCGGTC
Chromosome 6	chr6-TG-up	GAGCTGGAGCTACTGGTAATGACAAGGTC
	chr6-C-low	ATGATTGCTCAATGTAGGACCTGGCCTCAC
	Puro-L2	GCGAGGCGCACCGTGGGCTTGTACTCGGTC

Analyses

Usage	Primer name	Primer sequence
Quantitative RT-PCR	RTPCR-leader-F	GGACCGATCCAGCCTCCGCGGCC
	RTPCR-exon3-R	AGATACATTGTTATCTGAAGATG
	hActb-RTPCR-F	GCCGCGCTCGTCGTCGACAA
	hActb-RTPCR-R	CGATGGGGTACTTCAGGGTG
