# Supplementary material for: Generation of targeted homozygosity in the genome of human induced pluripotent stem cells
Source: PLoS One. 2019 Dec 5;14(12):e0225740. doi: 10.1371/journal.pone.0225740 (PMC6894808; doi:10.1371/journal.pone.0225740)
Supplement: S2 Table — (PDF) [file pone.0225740.s011.pdf]

S2 Table. Sequences of TALENs.

TALEN left:

GATCTACGCACGCTCGGCTACAGCCAGCAGCAACAGGAGAAGATCAAACCGAAGGTTCGTTCGACAGTGGCGCAGCACCACGAGGCAC
TGGTCGGCCATGGGTTTACACACGCGCACATCGTTGCGCTCAGCCAACACCCGGCAGCGTTAGGGACCGTCGCTGTCAAGTATCAGGA
CATGATCGCAGCGTTGCCAGAGGCGACACACGAAGCGATCGTTGGCGTCGGCAAACAGTGGTCCGGCGCACGCGCTCTGGAGGCCTTG
CTCACGGTGGCGGGAGAGTTGAGAGGTCCACCGTTACAGTTGGACACAGGCCAACTTCTCAAGATTGCAAAACGTGGCGGCGTGACCG
CAGTGGAGGCAGTGCATGCATGGCGCAATGCACTGACGGGTGCCCCCCTGAACCTGACTCCGGAACAGGTGGTGGCGATTGCAAGCAA
CATCGGCGGCAAACAGGCTCTAGAGACCGTGCAGCGCCTGCTGCCCGTGCTGTGCCAGGCCCACGGCCTGACCCCCGAGCAGGTGGTG
GCCATCGCCAGCAATAACGGCGGCAAGCAGGCGCTAGAGACCGTGCAGCGCCTGCTGCCCGTGCTGTGCCAGGCCCACGGCCTGACCC
CCGAGCAGGTGGTGGCCATCGCCAGCAATAACGGCGGCAAGCAGGCGCTAGAGACCGTGCAGCGCCTGCTGCCCGTGCTGTGCCAGGC
CCACGGCCTGACCCCCGAGCAGGTGGTGGCCATCGCCAGCAATATTGGCGGCAAGCAGGCGCTAGAGACCGTGCAGCGCCTGCTGCCC
GTGCTGTGCCAGGCCCACGGCCTGACCCCCGAGCAGGTGGTGGCCATCGCCAGCAATGGCGGCGGCAAGCAGGCGCTAGAGACCGTGC
AGCGCCTGCTGCCCGTGCTGTGCCAGGCCCACGGCCTGACCCCCGAGCAGGTGGTGGCCATCGCCAGCAATGGCGGCGGCAAGCAGGC
GCTAGAGACCGTGCAGCGCCTGCTGCCCGTGCTGTGCCAGGCCCACGGCCTGACCCCCGAGCAGGTGGTGGCCATCGCCAGCAATATT
GGCGGCAAGCAGGCGCTAGAGACCGTGCAGCGCCTGCTGCCCGTGCTGTGCCAGGCCCACGGCCTGACCCCCGAGCAGGTGGTGGCCA
TCGCCAGCAATGGCGGCGGCAAGCAGGCGCTAGAGACCGTGCAGCGCCTGCTGCCCGTGCTGTGCCAGGCCCACGGCCTGACCCCCGA
GCAGGTGGTGGCCATCGCCAGCAATAACGGCGGCAAGCAGGCGCTAGAGACCGTGCAGCGCCTGCTGCCCGTGCTGTGCCAGGCCCAC
GGCCTGACCCCCGAGCAGGTGGTGGCCATCGCCAGCAATAACGGCGGCAAGCAGGCGCTAGAGACCGTGCAGCGCCTGCTGCCCGTGC
TGTGCCAGGCCCACGGCCTGACCCCCGAGCAGGTGGTGGCCATCGCCAGCCACGACGGCGGCAAGCAGGCGCTAGAGACCGTGCAGCG
CCTGCTGCCCGTGCTGTGCCAGGCCCACGGCCTGACCCCCGAGCAGGTGGTGGCCATCGCCAGCAATGGCGGCGGCAAGCAGGCGCTA
GAGACCGTGCAGCGCCTGCTGCCCGTGCTGTGCCAGGCCCACGGCCTGACCCCCGAGCAGGTGGTGGCCATCGCCAGCAATAACGGCG
GCAAGCAGGCGCTAGAGACCGTGCAGCGCCTGCTGCCCGTGCTGTGCCAGGCCCACGGCCTGACCCCCGAGCAGGTGGTGGCCATCGC
CAGCCACGACGGCGGCAAGCAGGCGCTAGAGACCGTGCAGCGCCTGCTGCCCGTGCTGTGCCAGGCCCACGGCCTGACCCCCGAGCAG
GTGGTGGCCATCGCCAGCAATGGCGGCGGCAAGCAGGCGCTAGAGACCGTGCAGCGCCTGCTGCCCGTGCTGTGCCAGGCCCACGGCC
TGACCCCCGAGCAGGTGGTGGCCATCGCCAGCAATAACGGCGGCAAGCAGGCGCTAGAGACCGTGCAGCGCCTGCTGCCCGTGCTGTG
CCAGGCCCACGGCCTGACCCCCGAGCAGGTGGTGGCCATCGCCAGCAATGGCGGCGGCAAGCAGGCGCTAGAGACCGTGCAGCGCCTG
CTGCCCGTGCTGTGCCAGGCCCACGGCCTGACCCCCGAGCAGGTGGTGGCCATCGCCAGCAATGGCGGCGGCAAGCAGGCGCTAGAGA
GCATTGTTGCCCAGCTCTCCAGACCTGATCCGGCGCTAGCCGCGTTGCTAGTCAAAAGTGAACTCGAGGAGAAGAAATCTGAACTTCG
TCATAAATTGAAATATGTGCCTCATGAATATATTGAATTAATTGAAATTGCCAGAAATCCCACTCAGGATAGAATTCTTGAAATGAAG
GTAATGGAATTTTTTATGAAAGTTTATGGATATAGAGGTGAGCATTTGGGTGGATCAAGGAAACCGGACGGAGCAATTTATACTGTCG
GATCTCCTATTGATTACGGTGTGATCGTGGATACTAAAGCTTATAGCGGAGGTTATAATCTGCCAATTGGCCAAGCAGATGCCATGCA
AAGCTATGTCGAAGAAAATCAAACACGAAACAAACATATCAACCCTAATGAATGGTGGAAAGTCTATCCATCTTCTGTAACGGAATTT
AAGTTTTTATTTGTGAGTGGTCACTTTAAAGGAAACTACAAAGCTCAGCTTACACGATTAAATCATATCACTAATTGTAATGGAGCTG
TTCTTAGTGTAGAAGAGCTTTTAATTGGTGGAGAAATGATTAAAGCCGGCACATTAACCTTAGAGGAAGTGAGACGGAAATTTAATAA
CGGCGAGATAAACTTTCTCGAT

TALEN right:

GATCTACGCACGCTCGGCTACAGCCAGCAGCAACAGGAGAAGATCAAACCGAAGGTTCGTTCGACAGTGGCGCAGCACCACGAGGCAC
TGGTCGGCCATGGGTTTACACACGCGCACATCGTTGCGCTCAGCCAACACCCGGCAGCGTTAGGGACCGTCGCTGTCAAGTATCAGGA
CATGATCGCAGCGTTGCCAGAGGCGACACACGAAGCGATCGTTGGCGTCGGCAAACAGTGGTCCGGCGCACGCGCTCTGGAGGCCTTG
CTCACGGTGGCGGGAGAGTTGAGAGGTCCACCGTTACAGTTGGACACAGGCCAACTTCTCAAGATTGCAAAACGTGGCGGCGTGACCG
CAGTGGAGGCAGTGCATGCATGGCGCAATGCACTGACGGGTGCCCCCCTGAACCTGACTCCGGAACAGGTGGTGGCGATTGCAAGCAA
CAACGGCGGCAAACAGGCTCTAGAGACCGTGCAGCGCCTGCTGCCCGTGCTGTGCCAGGCCCACGGCCTGACCCCCGAGCAGGTGGTG
GCCATCGCCAGCAATATTGGCGGCAAGCAGGCGCTAGAGACCGTGCAGCGCCTGCTGCCCGTGCTGTGCCAGGCCCACGGCCTGACCC
CCGAGCAGGTGGTGGCCATCGCCAGCCACGACGGCGGCAAGCAGGCGCTAGAGACCGTGCAGCGCCTGCTGCCCGTGCTGTGCCAGGC
CCACGGCCTGACCCCCGAGCAGGTGGTGGCCATCGCCAGCAACAACGGCGGCAAGCAGGCGCTAGAGACCGTGCAGCGCCTGCTGCCC
GTGCTGTGCCAGGCCCACGGCCTGACCCCCGAGCAGGTGGTGGCCATCGCCAGCAATGGCGGCGGCAAGCAGGCGCTAGAGACCGTGC
AGCGCCTGCTGCCCGTGCTGTGCCAGGCCCACGGCCTGACCCCCGAGCAGGTGGTGGCCATCGCCAGCAATGGCGGCGGCAAGCAGGC
GCTAGAGACCGTGCAGCGCCTGCTGCCCGTGCTGTGCCAGGCCCACGGCCTGACCCCCGAGCAGGTGGTGGCCATCGCCAGCCACGAC
GGCGGCAAGCAGGCGCTAGAGACCGTGCAGCGCCTGCTGCCCGTGCTGTGCCAGGCCCACGGCCTGACCCCCGAGCAGGTGGTGGCCA
TCGCCAGCAATGGCGGCGGCAAGCAGGCGCTAGAGACCGTGCAGCGCCTGCTGCCCGTGCTGTGCCAGGCCCACGGCCTGACCCCCGA
GCAGGTGGTGGCCATCGCCAGCAATATTGGCGGCAAGCAGGCGCTAGAGACCGTGCAGCGCCTGCTGCCCGTGCTGTGCCAGGCCCAC
GGCCTGACCCCCGAGCAGGTGGTGGCCATCGCCAGCAACAACGGCGGCAAGCAGGCGCTAGAGACCGTGCAGCGCCTGCTGCCCGTGC
TGTGCCAGGCCCACGGCCTGACCCCCGAGCAGGTGGTGGCCATCGCCAGCAATGGCGGCGGCAAGCAGGCGCTAGAGACCGTGCAGCG
CCTGCTGCCCGTGCTGTGCCAGGCCCACGGCCTGACCCCCGAGCAGGTGGTGGCCATCGCCAGCAATGGCGGCGGCAAGCAGGCGCTA
GAGACCGTGCAGCGCCTGCTGCCCGTGCTGTGCCAGGCCCACGGCCTGACCCCCGAGCAGGTGGTGGCCATCGCCAGCAACAACGGCG
GCAAGCAGGCGCTAGAGACCGTGCAGCGCCTGCTGCCCGTGCTGTGCCAGGCCCACGGCCTGACCCCCGAGCAGGTGGTGGCCATCGC
CAGCCACGACGGCGGCAAGCAGGCGCTAGAGACCGTGCAGCGCCTGCTGCCCGTGCTGTGCCAGGCCCACGGCCTGACCCCCGAGCAG
GTGGTGGCCATCGCCAGCAATGGCGGCGGCAAGCAGGCGCTAGAGACCGTGCAGCGCCTGCTGCCCGTGCTGTGCCAGGCCCACGGCC
TGACCCCCGAGCAGGTGGTGGCCATCGCCAGCCACGACGGCGGCAAGCAGGCGCTAGAGACCGTGCAGCGCCTGCTGCCCGTGCTGTG
CCAGGCCCACGGCCTGACCCCCGAGCAGGTGGTGGCCATCGCCAGCCACGACGGCGGCAAGCAGGCGCTAGAGACCGTGCAGCGCCTG
CTGCCCGTGCTGTGCCAGGCCCACGGCCTGACCCCCGAGCAGGTGGTGGCCATCGCCAGCAATGGCGGCGGCAAGCAGGCGCTAGAGA
CCGTGCAGCGCCTGCTGCCCGTGCTGTGCCAGGCCCACGGCCTGACCCCCGAGCAGGTGGTGGCCATCGCCAGCAACAACGGCGGCAA
GCAGGCGCTAGAGACCGTGCAGCGCCTGCTGCCCGTGCTGTGCCAGGCCCACGGCCTGACCCCCGAGCAGGTGGTGGCCATCGCCAGC
AATGGCGGCGGCAAGCAGGCGCTAGAGAGCATTGTTGCCCAGCTCTCCAGACCTGATCCGGCGCTAGCCGCGTTGCTAGTCAAAAGTG
AACTCGAGGAGAAGAAATCTGAACTTCGTCATAAATTGAAATATGTGCCTCATGAATATATTGAATTAATTGAAATTGCCAGAAATCC
CACTCAGGATAGAATTCTTGAAATGAAGGTAATGGAATTTTTTATGAAAGTTTATGGATATAGAGGTGAGCATTTGGGTGGATCAAGG
AAACCGGACGGAGCAATTTATACTGTCGGATCTCCTATTGATTACGGTGTGATCGTGGATACTAAAGCTTATAGCGGAGGTTATAATC
TGCCAATTGGCCAAGCACGAGAAATGCAACGATATGTCGAAGAAAATCAAACACGAAACAAACATATCAACCCTAATGAATGGTGGAA
AGTCTATCCATCTTCTGTAACGGAATTTAAGTTTTTATTTGTGAGTGGTCACTTTAAAGGAAACTACAAAGCTCAGCTTACACGATTA
AATCATATCACTAATTGTAATGGAGCTGTTCTTAGTGTAGAAGAGCTTTTAATTGGTGGAGAAATGATTAAAGCCGGCACATTAACCT
TAGAGGAAGTGAGACGGAAATTTAATAACGGCGAGATAAACTTTCTCGAT
