# Supplementary material for: Generation of targeted homozygosity in the genome of human induced pluripotent stem cells
Source: PLoS One. 2019 Dec 5;14(12):e0225740. doi: 10.1371/journal.pone.0225740 (PMC6894808; doi:10.1371/journal.pone.0225740)
Supplement: S3 Table — (PDF) [file pone.0225740.s012.pdf]

S3 Table. CRISPR information.

Name of CRISPR	SNP ID	SNP type	gRNA targeting sequence
9M	rs2965273	A/G	CCTGGTGTGGGGGCCCTGCG
14M	rs17656487	C/T	GTCAGGGATGGGATTAGGGA
19M-I	rs12977652	A/G	CCCAGAAGCCTCCGCGGCGC
19M-II	rs1727740	C/T	ATTGTTATATTGGTGAGGGG
HLA	rs2246618	C/T	ATTGCTTTGATGCTGGGTCA
