# Supplementary material for: Generation of targeted homozygosity in the genome of human induced pluripotent stem cells
Source: PLoS One. 2019 Dec 5;14(12):e0225740. doi: 10.1371/journal.pone.0225740 (PMC6894808; doi:10.1371/journal.pone.0225740)
Supplement: S4 Table — (PDF) [file pone.0225740.s013.pdf]

S4 Table. Primers used for SNP typing.

Chromosome 19_9M region

Name	SNP ID	Primer name	Primer sequence	Primer name	Primer sequence
At 8Mb	rs12976422	8M_F	GCCTCATGTCAGTGTCAGAGATACAGTGGC	8M_R	AAGGGGACTGTTTCTCCTGGGGGTGGTCTC
9M−64kb	rs3787034	9M−64kb_F	GATCAGTGGAATAAAAAATGGTACCTGG	9M−64kb_R	CAGCCATTTCTATCAAAATATACTTAATCC
9M−22kb	rs4321301	9M−22kb_F	GCCCTTGAATCCTCAGACAGCAGCAGCGGC	9M−22kb_R	GGGTGTAAAATTATGATCAAAAATAAATTG
9M−17kb	rs35570240	9M−17kb_F	GCCTCCAGTCCCCACATGCTCATCGTGTGTG	9M−17kb_R	TGGGGTAGACGCCATAAAATAGGTGAGGCT
9M−8kb	rs12973037	9M−8kb_F	GTCCCTGTCATCCTTCCCTGGTCTGAG	9M−8kb_R	CCAAGAGCAGTTCCACTTGGGATTGTC
9Mb	rs2965273	9M_F	GTCAGGGTCAGACTCCACCTTGAGTCTGGC	9M_R	GGTAGTTGAGGAACCACGTGTGTATTGGGT
9M+24kb	rs113292307	9M+24kb_F	GAGCATATCTCTTGGTTGGGTCTGTGTG	9M+24kb_R	CAGAGGACTACAGGTCTTCAGCATCATC
9M+200kb	rs11668379	9M+200kb_F	GAAAGATCAGCAAACACCACGTGCAGCAAC	9M+200kb_R	TTCTTGAGGAGAATGTCTTCCGCTGTTGGG
9M+300kb	rs1345654	9M+300kb_F	AAGCAAAACGTACTTTTATATAACCAATGGGTC	9M+300kb_R	AGAATGAATTAGGAAAAGTTGCCTCCTTCT
9M+340kb	rs8106116	9M+340kb_F	GATTTTGCTTCTTAGGCACTGAGTTCCTGG	9M+340kb_R	ATCAAGATAAAGGGCCACTGCAGCAGTGCC
9M+1Mb	rs11882238	9M+1Mb_F	CAGGAGGCTGGGGCCCATGACAGCCTTGGG	9M+1Mkb_R	TTGCCATTGAGCCTGCTCCCCTCAATGGGG

Chromosome 19_14M region

Name	SNP ID	Primer name	Primer sequence	Primer name	Primer sequence
14M−30kb	rs4803341	14M−30kb_F	GGCTCGCAGGACACCCCTTCCCGGCTTCCC	14M−30kb_R	TGTCACACACACACAAAAGGGTAGGCCAGG
14M−2kb	rs10414006	14M−2kb_F	CACACAGACATAAAGACCCATCCCTTCACC	14M−2kb_R	AATAGCACCTGCCCATAGGTGGATCGGAC
14Mb	rs17656487	14M_F	CCCTGCTTTCAACCATCTCCCACTAAACCA	14M_R	AAGGGAGCAGCTTTAATTTGGGATGGGAGA
14M+6kb	rs7257372	14M+6kb_F	GAGAAATAGAAACATGCTGGACACGGTGGC	14M+6kb_R	CTTGTCTCATCTCTCTCTAGATCTGGTAGG
14M+90kb	rs12977500	14M+90kb_F	AGATCCTGGCTCTGGAGTCGGCAGATGGGC	14M+90kb_R	CCATCCCACCCCGACCTCTCCCCCTGGCC

Chromosome 19_19M region

Name	SNP ID	Primer name	Primer sequence	Primer name	Primer sequence
19M–I–35kb	rs630473	19M−I−35kb_F	TGCTGGTTTTGGTTAGCTTGGGTATGTCCT	19M−I−35kb_R	TGGTTTCTACGACAAGTCAACAGCAGGAGA
19M–I–6kb	rs13345771	19M−I−6kb_F	CAGGATGTTTTTGCTTTCCCTATGTA	19M−I−6kb_R	TGGGAGAGGAACTACCTACAGGGGGC
19M–I	rs12977652	19M−I_F	CCAGAGCCACCTGCTCGGAATCTTACCTAC	19M−I_R	CCCCGCCATTTCTATTGGATTCTGACGTCA
19M−II−16kb	rs12982449	19M−II−16kb_F	CTAGAAGGAAGAACTTAAAGAGAAGGAGG	19M−II−16kb_R	CTTGTGGGCCCCACAAGGGCAGGAGATGAG
19M−II−8kb	rs13345724	19M−II−8kb_F	CTCTGTGATACTCCAACTCTCTCCCCGCG	19M−II−8kb_R	CCTTAGAAATGATACCAGTGGGGCCCAGGC
19M−II−2kb	rs1727733	19M−II−2kb_F	GCTTTTAAAAAATTTAAGAATGCAGA	19M−II−2kb_R	CAGAAGTGAATTAATTCTTAGAAACCTCC
19M–II	rs1727740	19M−II_F	GCAGCTGAATGCATTCTACCTGGCTCCT	19M−II_R	CTAATTGGTGGTGTCCCAAGTTCTAGAAG

Chromosome 6

Name	SNP ID	Primer name	Primer sequence	Primer name	Primer sequence
HLA−12kb	rs9267464	HLA−12kb_F	CCAAGAAATAGTCCCACATAAATACAAGGA	HLA−12kb_R	GACTAAGACACATAGATTATGTTACTGTG
HLA−5kb	rs9267444	HLA−5kb_F	CCTGATCAGATTGAGCCAGGACAATGGCCG	HLA−5kb_R	ACATGGCAAGGTGTGGTTGCCCTCAGGGTC
HLA	rs2246618	HLA_F	CTTGTCACCTTGAGGTCCCCTCGCCCCGTC	HLA_R	CAGGCCGGATCACAGCCAGAACCTCCTCCC
