# Supplementary material for: Generation of targeted homozygosity in the genome of human induced pluripotent stem cells
Source: PLoS One. 2019 Dec 5;14(12):e0225740. doi: 10.1371/journal.pone.0225740 (PMC6894808; doi:10.1371/journal.pone.0225740)
Supplement: Attachment — Submitted filename: Response to the Reviewer 1007.docx [file pone.0225740.s014.docx]

**Response to the Reviewer 1**

**1, It would be interesting analyze more independent clones (only 2 clones were analyzed) for experiments on HLA (Figure4).**

We appreciate the Reviewer’s valuable comment.

We previously analyzed additional independent clones and obtained a clone with G418 and puromycin double resistance. The pattern of the crossing over in the HLA locus was the same as that in #1 and, thus, we had previously omitted it. In accordance with the reviewer’s suggestion, we have added this result in Fig 4C.

**2, It would have been interesting to measure whether pluripotency markers (POU5F1, NANOG, alkaline phosphatase activity…) are affected in manipulated cells.**

We agree with the reviewer’s comment.

We have now shown additional data of the expression of pluripotency marker genes in S8 Fig. We have also added a sentence starting at line 166

**3, If I am right, the double selection requires an inversion of the selection cassette and the use of a CRE. You mention that you used iCRE but I have not understood well what dives the expression of the CRE and how efficient it is. Could you please comment on that and be more explicit?**

We appreciate the reviewer’s comment.

To clarify iCRE, we have explained how iCRE was generated starting at line 108.

We have also added a sentence in the “Methods” section, indicating that the expression of iCRE was driven by the EF1α promoter starting at line 236.

**4, Why starting the puro treatment before G418.**

Because we wanted to eliminate hiPSC clones that did not undergo Cre-mediated inversion, we started puro selection before the double selection. This sentence has been added starting at line 112.

**5, Even if out of scope of this study it will be interesting to know whether LOH occurs in other unwanted DNA regions. The selection cassette permits to select for positive clones but this does not mean that other regions are not affected due to over recombination?**

We appreciate your very important comments that irrelevant recombinations might occur in many different places of the human genome with our strategy. We have previously performed SNP array analysis to estimate irrelevant recombination, but we did not include these data in the previous manuscript.

These data have been now shown in the revised manuscript in S7 Fig. Although chromosomal abnormalities were observed in chromosomes 3 and 4, it might have occurred during hiPSC generation. There were no irrelevant crossovers in CRISPR/Cas9-manipulated hiPS cells. We have described this result starting at line 153.

**6, Line 443. It is mentioned that DSBs were either induced by CRISPR/Cas9 or TALEN. I thought that authors had only used CRISPR/Cas9?**

Thank you for your comments.

We have omitted TALEN in the Fig 1 legend.

**7, In fig. 2C, clone 25, which behaves very differently from all other clones, is marked by an * but this particular clone is not commented.**

Thank you for your comments.

This clone was probably derived from uniparental disomy or a spontaneous DSB close to the centromere, which was independent of the DSB induced by CRISPR/Cas9. This possibility has been mentioned in the “Results” starting at line 131.

**8 Line 456. There are legends for panel E and F but these panels are missing in the figure.**

Thank you for your comment.

We have deleted E and F from the Fig. 2 legend.

**9. Line 461. Change ‘suppression’ for inhibition as ML216 inhibits DNA binding and blocks BLM helicase activity, protein suppression let think that the protein is destroyed.**

Thank you for your comments.

In accordance with the reviewer’s suggestion, we have replaced ‘suppression’ with ‘inhibition’ in the Fig 3 legend.

**Response to the Reviewer 2**

**1, The abstract does not provide a clear idea of the importance of the study. It starts suggesting that LOH can be used to identify genes associated with loss or gain of disease phenotype, but after describing the approach, it says that the system is useful in regenerative medicine. In this regard, it is clear that the approach is useful as a tool for genetic analysis, but in this reviewer’s opinion, the authors may want to reconsider suggesting its use in regenerative medicine as chromosomal crossover can cause genetic instability and it has been associated with cancer.**

In accordance with reviewer’s suggestion, we have modified the “Abstract”.

**2. In the abstract, authors claim that ML216 treatment had similar crossover efficiency than Tet-Off-driven BLM suppression but there is no actual data to compare “efficiency”. Instead, both approaches successfully promoted crossover.**

We have added the actual data of crossover efficiency with ML216 treatment in Fig 3B.

We have also added a sentence in the “Results” section starting at line 149.

**3. In author summary, authors once again suggest that chromosomal crossover might be useful for stem cell-based therapy. Consider revising this, otherwise authors should include in discussion association of crossovers with cancer or other potential limitations of the approach.**

We appreciate the reviewer’s valuable comments.

In accordance with the reviewer’s suggestion, we have rewritten the “Author summary” and “Discussion” starting at line 201.

**4. Introduction does not state clearly the relevance of the study or the utility of the approach. Instead, authors claim that it has been tested in other cell types/species but not in hiPSCs.**

In accordance with reviewer’ s suggestion, we have rewritten the “Introduction”.

**5. In line 211, authors mention that the CMV promoter was changed to hEF1a but the diagram on Fig. 1B still shows the CMV promoter.**

Thank you for your comments.

Because Fig 1B is confusing, it has been redrawn.

We have also modified the vector construction in the “Methods” section starting at line 217.

**5. It is not mentioned whether tTA is expressed under the native BLM promoter. If this is the case, it makes sense that BLM expression in parental and BLMtet/tet lines (No Dox) is similar. However, since this cassette is largely used in the study, it would be important to include this expression comparison.**

Thank you for your valuable comments.

A comparison of BLM expression between parental cells and the Tet cassette-inserted cell line is now shown S2 Fig.

**7. Authors do not show experimental evidence on the removal of Pgk-Puro cassette used to select BLMtet/tet clones, this is critical as the authors rely on an additional positive Puro selection to identify crossover.**

Thank you for your valuable comments.

We have represented these data in new S1 Fig. A sentence has been added starting at line 72.

**7, (6). In line 91, authors describe that “Upon Cre expression, inversion of the cNP cassette occurred, resulting in loss of G418 resistance and gain of puromycin”, however, no experimental data are shown. This is a constant along the manuscript that needs to be addressed. This is important as the reader can get an idea of the efficiency of the system to induce inversion of the selection cassette.**

Thank you for your valuable comments.

We have added new S4 Fig to show the estimated efficiency of the inversion of the selection cassette by iCre. We have also added a sentence starting at line 85.

**8, (7). In Fig. 1B, authors show a bar graph in which about 3 clones out of 1e6 cells were positive for the double (Puro/Neo) selection. Although this suggests that crossover was successfully achieved, the authors should comment on the low efficiency of the approach, which might be explained by all the variables in the system (e.g. Dox incubation, DSB in 4n phase etc).**

Thank you for your valuable comments.

We have added a sentence explaining why the number of colonies was low in the “Results” section starting at line 121.

**9,(8). There is no explanation on why the authors performed the experiments after 7 days with Dox (only two time points were analyzed for BLM expression, 5d and 7d).**

Thank you for your valuable comment.

In fact, we analyzed BLM expression after 8 days of dox treatment. These data are shown in S3 Fig. hiPSCs with the Tet cassette appeared to be unhealthy with prolongation of dox exposure. Thus, we chose 7 days of treatment with dox. This sentence has been added starting at the line 77.

**10, (9). In line 130, authors mention that when crossover occured centromeric to DSBs, the distance between the crossovers and DSBs was small. Then, a similar pattern is observed at 14Mb from centromere. However, according to Fig.2C, it seems that only one clone out of 25 resulted in crossover centromeric to the DSB (and one out of six in Fig. S2B). Are these numbers sufficient to conclude a “pattern” of centromeric vs telomeric crossover proximity?**

Thank you your valuable comment.

We have added a new sentence to clarify that each colony was derived from different clones in the “Results” section starting at line 129.

**11, (10). Figure 2 legend has (E) and (F) but this is not shown in the figure.**

Thank you for your comments

We have deleted E and F from the Fig 2 legend.

**12, (11). The authors do not include any data on how effective the ML216 treatment is in their experimental setting.**

We have added the actual data of crossover efficiency with ML216 treatment in Fig 3B.

**12. Materials and methods say that ML216 was used 12h before transfection but figure legend (line 462) says that treatment began 24h before transfection.**

In fact, 12 h is correct. Thus, we have changed 1 day to 12 h in the Fig 3A legend.

**13. Although a similar pattern of SNPs is observed between Tet-off and ML216, there is no data on the efficiency of ML216 treatment (how many positive clones?).**

We have added the actual data of crossover efficiency with ML216 treatment in Fig 3B.

**14. Fig 3C hiPS-BLMtet/tetAAVS1cNP/+ and Fig. 2D (same condition) are identical. If the same SNP analysis in Fig 2D was used as a reference for Fig 3C, it should be mentioned in the text to avoid misleading interpretation of intentional image duplication. Ideally, however, each assay (Fig 2D and 3C) should have been run in parallel with its own negative control.**

We appreciate the important comment.

In accordance with the reviewer’s suggestion, we have modified Fig 3C.

**15. In Fig. 3, ML216 treatment is used in cells that have the BLMtet/tet cassette. Again, showing that cassette insertion (No Dox) does not modify itself BLM expression would avoid concerns on the cassette background.**

We have added the actual data of BLM expression in parental and cassette-inserted cells in S2 Fig.

**16. In Fig.4C, directionalities of the chromosome, DSB and crossover are confusing. The chromosome diagram shows that Tel is top and Cent is bottom, while this seems to be the opposite in the DSB diagram next to it?**

Thank you for your careful observation.

In accordance with the reviewer’s suggestion, we have redrawn Fig 4C.

**17. Fig. 4E shows parental cells positive for HLA-A32 and HLA-A2. However, it would be more important to show the cells that were edited to include the selected cassette but did not have ML216 treatment (negative control). Furthermore, authors claim that these cells are HLA-homozygous hiPSCs without exogenous genetic elements (line 180) while no data show that the selection cassette was removed.**

We appreciate your valuable comment.

We have added the flow cytometry profile of control cells in S9 Fig. We have also added a sentence starting at line 170.

Our discussion was incorrect. We did not establish HLA-homozygous hiPSCs without exogenous genetic elements Thus, we have rewritten the “Discussion” section starting at line 184.

**18. It would be useful to see whether the hiPSCs retained pluripotency after chromosomal crossover and all the genetic editing that they were subjected to.**

We agree with the reviewer’s comment.

We have now shown additional data of the expression of pluripotency marker genes in S8 Fig. We have added a sentence starting at line 166.

**19. Other experimental approaches such as FISH or Southern blot would provide more solid evidences on the crossover efficiency, cassette integration/removal, transposon removal etc.**

We agree with the reviewer’s comment.

To check whether unwanted events occurred in the human genome, we have added genome-wide SNP array data in S7 Fig. We have also added a sentence starting at line 153.
