# Supplementary material for: Generation of targeted homozygosity in the genome of human induced pluripotent stem cells
Source: PLoS One. 2019 Dec 5;14(12):e0225740. doi: 10.1371/journal.pone.0225740 (PMC6894808; doi:10.1371/journal.pone.0225740)
Supplement: Attachment — Submitted filename: Response to the Reviewer 1105.docx [file pone.0225740.s015.docx]

Response to the Reviewer 2

1. **Supplementary Figure 4 legend does not explain what the blue staining means, which is essential to underatand the iCre-mediated cassette inversion selection.**

We appreciate the Reviewer’s valuable comment. We added a new sentence in S4 Fig legend starting at line 449.

**2. In line 88, authors claim the efficiency of inversion was 20%-30%, but it is not clear what they measured to obtain this number.**

We appreciate the Reviewer’s valuable comment. We now added a new sentence starting at line 87.

3. **In line 134, revise “we established a two more DSBs” for “we established two more DSBs”.**

We modified a sentence starting at line 134**.**

4. **In S6 Fig A-C consider changing M for Mb to be consistent with text (eg 9Mb, 14Mb, 19Mb-I).**

We modified S6 Fig A-C.

5.  **In line 157, the authors start describing the generation of HLA-homozygous iPSC. This seems out of context since the rational of doing it is not mention in the introduction nor in the results section. Maybe a few sentences describing why the authors were interested on achieving HLA homozygosity in hiPSCs would make it easier for the reader to understand this section.**

Thank you for the Reviewer’s valuable comment. We added new sentences starting at line 158.
